# Supplementary figures and images for: Genome Wide Identification and Characterization of Wheat GH9 Genes Reveals Their Roles in Pollen Development and Anther Dehiscence
Source: Int J Mol Sci. 2022 Jun 5;23(11):6324. doi: 10.3390/ijms23116324 (PMC9181332; doi:10.3390/ijms23116324)

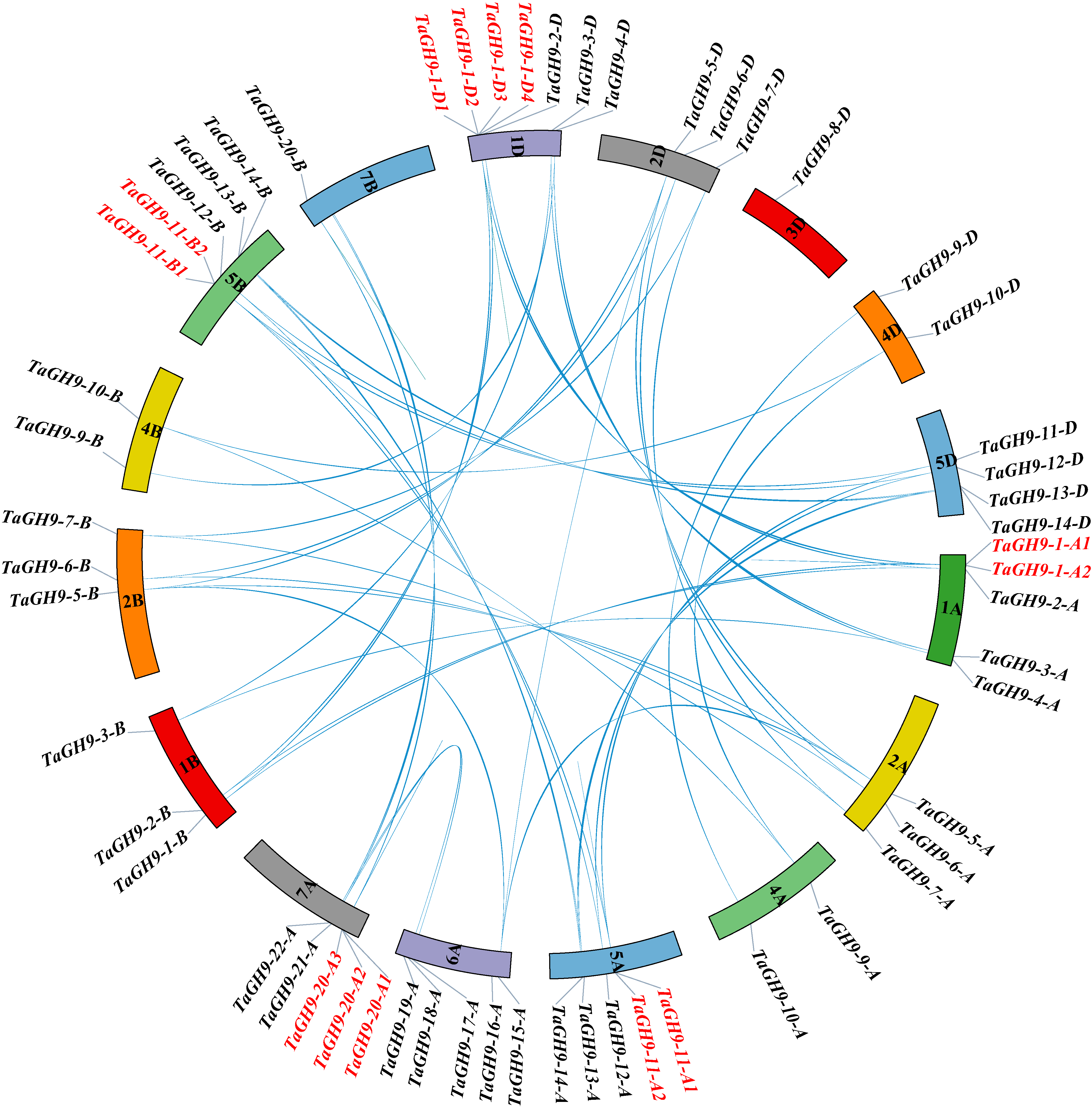

Supplement: Supplementary file 1 [file ijms-23-06324-s001.zip › Supplementary Materials/Figure S2.tif]

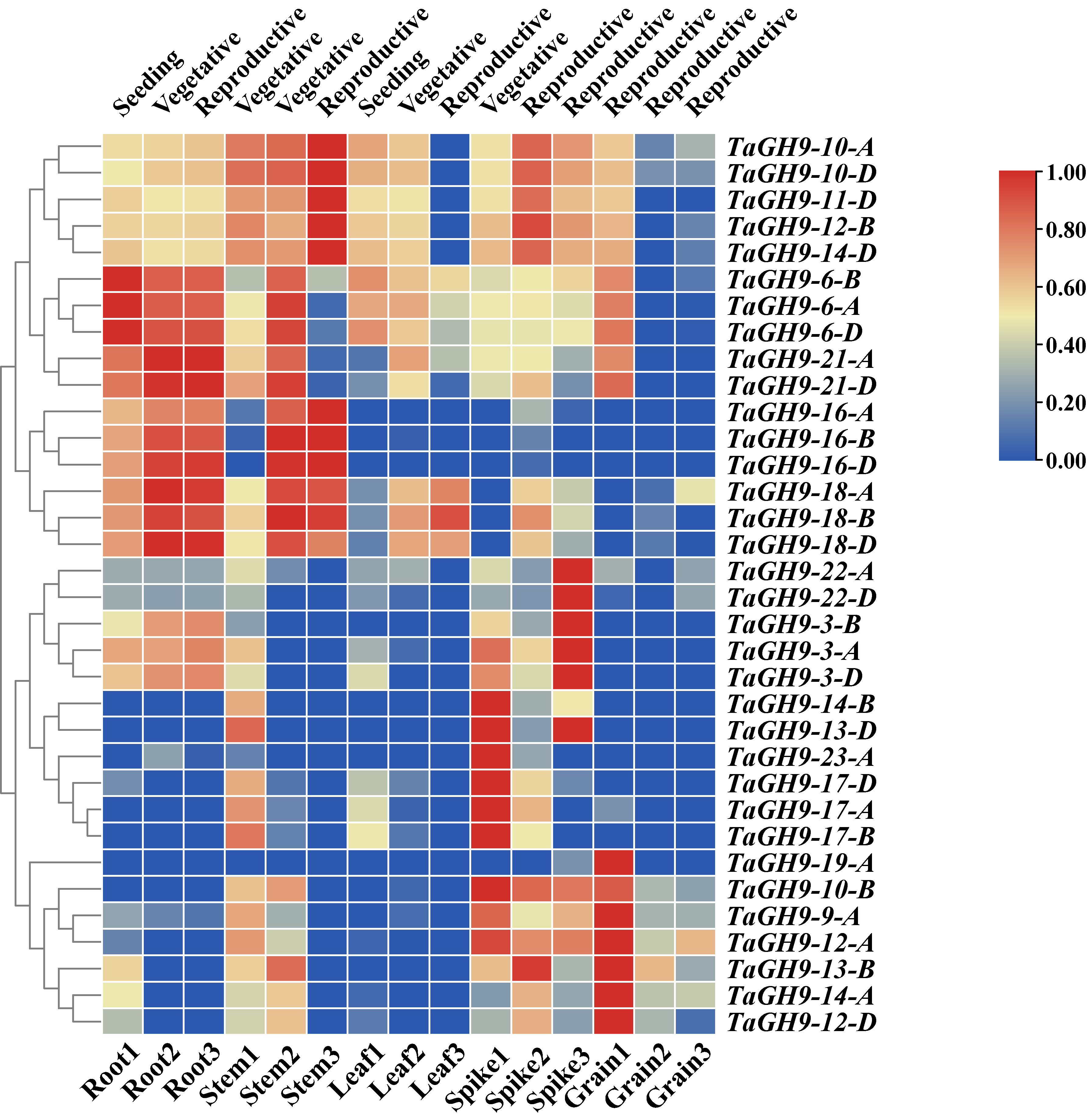

Supplement: Supplementary file 1 [file ijms-23-06324-s001.zip › Supplementary Materials/Figure S4.tif]
